# Different Estimation Procedures for the Parameters of the Extended Exponential Geometric Distribution for Medical Data

**DOI:** 10.1155/2016/8727951

**Published:** 2016-08-04

**Authors:** Francisco Louzada, Pedro L. Ramos, Gleici S. C. Perdoná

**Affiliations:** ^1^Statistics Department, Institute of Mathematical and Computer Sciences (ICMC), São Paulo University (USP), 13560-970 São Carlos, SP, Brazil; ^2^Department of Social Medicine, Ribeirão Preto School of Medicine (FMRP), São Paulo University (USP), 14049-900 Ribeirão Preto, SP, Brazil

## Abstract

We have considered different estimation procedures for the unknown parameters of the extended exponential geometric distribution. We introduce different types of estimators such as the maximum likelihood, method of moments, modified moments,* L*-moments, ordinary and weighted least squares, percentile, maximum product of spacings, and minimum distance estimators. The different estimators are compared by using extensive numerical simulations. We discovered that the maximum product of spacings estimator has the smallest mean square errors and mean relative estimates, nearest to one, for both parameters, proving to be the most efficient method compared to other methods. Combining these results with the good properties of the method such as consistency, asymptotic efficiency, normality, and invariance we conclude that the maximum product of spacings estimator is the best one for estimating the parameters of the extended exponential geometric distribution in comparison with its competitors. For the sake of illustration, we apply our proposed methodology in two important data sets, demonstrating that the EEG distribution is a simple alternative to be used for lifetime data.

## 1. Introduction

Many researches are interested in search distributions which can be used to describe real data sets. Generalizations of the standard exponential distribution have been introduced in the literature for this purpose, such as Gamma, Weibull, and Generalized Exponential distribution [1]. Another useful generalization is known as extended exponential geometric distribution. Initially, the development of such distribution was made by Adamidis and Loukas [2] proposing exponential geometric distribution with two parameters, in which the hazard function could be decreasing. In a further paper, Adamidis et al. [3] explored extended exponential geometric (EEG) distribution. Let *X* be a random variable representing a lifetime data, with extended exponential geometric (EEG) distribution; its probability density function (PDF) is given by

(1) $$\begin{array}{c} f\left(\;x\;|\;\gamma ,\lambda \;\right)=\frac{\lambda \gamma e^{-\lambda x}}{{\left(1-\left(1-\gamma \right)e^{-\lambda x}\right)}^{2}}, \end{array}$$

for all *x* > 0, *γ* > 0, and *λ* > 0. One of its peculiarities is that its hazard function can be increasing or decreasing, depending on the values of its parameters, giving great flexibility of fit for real applications.

This model arises naturally in competing risks scenarios. Let *X* = min⁡(*T*_1_, *T*_2_,…, *T*_*M*_), where* M* is a random variable with geometrical distribution and *T*_*i*_ are independent of* M* and are assumed to be independent and identically distributed according to exponential distribution; then the random variable *X* has EEG distribution with 0 < *γ* < 1, also known as exponential geometric (EG) distribution [2]. Considering the same assumptions and *X* = max⁡(*T*_1_, *T*_2_,…, *T*_*M*_), the random variable *X* has EEG distribution with *γ* > 1, also known as Complementary Exponential Geometric distribution [4]. Due to its importance, some generalizations of the EEG distribution have been proposed, such as the Beta exponential geometric distribution [5], Exponentiated Exponential-Geometric distribution [6], Complementary Exponentiated Exponential Geometric distribution [7], and Generalized Exponential Geometric distribution [8].

Despite the fact that EEG distribution has good flexibility, a few estimation procedures have been proposed in the literature. Adamidis et al. [3] derived the maximum likelihood estimators (MLE) for the unknown parameters of the EEG distribution. Ramos et al. [9] developed a Bayesian analysis under noninformative priors. However, considering the frequentist approach, it is well known that, usually, for small samples, the MLE does not perform well. In this paper, we proposed nine new estimators for the parameters of the EEG distribution, which are given considering the following estimation procedures: the method of moments, modified moments, ordinary least squares, weighted least squares,* L*-moments, percentile, maximum product of spacings, Cramer-von Mises type minimum distance, and Anderson-Darling estimator.

The main aim of this paper is twofold. First, it aims to develop a guideline for choosing the most efficient estimators among ten different estimation procedures for the EEG distribution, which would be of interest to applied statisticians. Second, it aims to demonstrate that the EEG distribution is a simple alternative to be used in applications in medicine.

The originality of this study comes from the fact that, for the EEG distribution and considering the frequentist approach, only the MLE has been presented in the literature. The performances of the different estimation methods are compared using extensive numerical simulations. Additionally, these results are analogous for the exponential geometric distribution and the Complementary Exponential Geometric distribution. Related studies for other distributions can be found in Gupta and Kundu [10], Mazucheli et al. [11], Teimouri et al. [12], and Dey et al. [13].

The paper is organized as follows. In Section 2, we discuss some properties of the EEG distribution. In Section 3, we present ten estimation procedures for the parameters of our proposed model. In Section 4, a simulation study is presented in order to identify the most efficient estimators. In Section 5, we apply our proposed methodology in two real data sets. Some final comments are presented in Section 6.

## 2. Extended Exponential Geometric Distribution

Let *X* be a random variable with density function (1); the distribution function is given by

(2) $$\begin{array}{c} F\left(\;x\;|\;\gamma ,\lambda \;\right)=\frac{1-e^{-\lambda x}}{1-\left(1-\gamma \right)e^{-\lambda x}}. \end{array}$$

The survival and hazard functions of EEG(*γ*, *λ*) distribution is given, respectively, by

(3) Sx ∣ γ,λ=γe−λx1−1−γe−λx,hx ∣ γ,λ=λ1−1−γe−λx.

**Figure 1:**
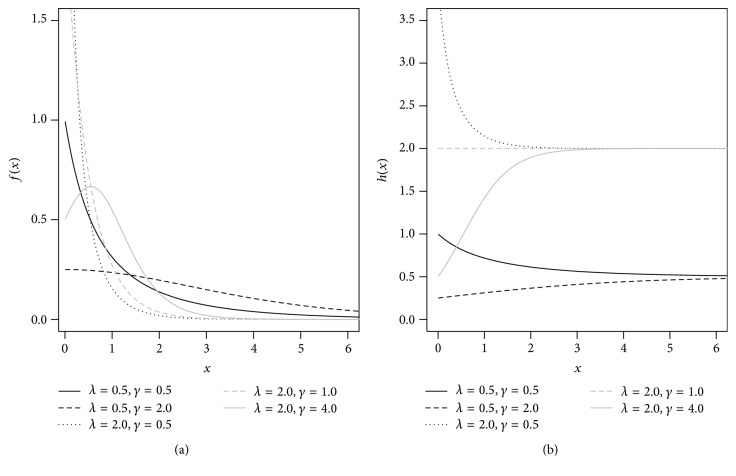
(a) Probability density function of the EEG distribution. (b) Hazard function of the EEG distribution.

The hazard function (3) is decreasing for 0 < *γ* < 1, is constant for *γ* = 1, and is monotonically increasing when *γ* > 1.  Figure 1 presents different forms for the density and hazard functions for the EEG distribution considering different values of *γ* and *λ*.

For the random variable *X* with EEG distribution, the moment generating function [14] is given by

(4) $$\begin{array}{c} M_{X}\left(\;t\;\right)=1+\frac{t\gamma }{\lambda }\Phi \left(\;1-\gamma ,1,1-\frac{t}{\lambda }\;\right), \end{array}$$

for *t* < *λ*, where Φ(*z*, *s*, *a*) = Γ(*s*)^−1^∫_0_^*∞*^ *t* ^*s*−1^ *e* ^−*at*^(1 − *ze* ^−*t*^)^−1^ *dt*, for *a*, *s* > 0, and *z* < 1 is known as Lerch transcendental function [15]. Note that the Laplace transform of the EEG distribution can be easily obtained from the relation LT_*X*_(*t*) = *M*_*X*_(−*t*) = *E*(*e* ^−*tX*^). The raw moments of the EEG distribution are

(5) $$\begin{array}{c} E\left(\;X^{r}\;|\;\gamma ,\lambda \;\right)=r!\gamma \lambda^{-r}\Phi \left(\;1-\gamma ,r,1\;\right), \end{array}$$

for *r* ∈ *N*. After some algebraic manipulation, the mean and variance of the EEG distribution are given, respectively, by

(6) EX ∣ γ,λ=γlog⁡γλγ−1,VarX ∣ γ,λ=γλ22L21−γ1−γ−γ log2⁡γ1−γ2,

where *L*_2_(*z*) is the dilogarithm function given by

(7) L2z∑k=1∞zkk2=−∫0zlog⁡1−ttdt=−∫01log⁡1−zttdt.

The mode and the median of the EEG distribution are

(8) ModeX ∣ γ,λ=0if  γ≤2log⁡γ−1λif  γ≥2,MedianX ∣ γ,λ=log⁡1+γλ.

From Marshall and Olkin [16], we have the following inequality:

(9) ModeX ∣ γ,λMedianX ∣ γ,λ≤γλ≤EX ∣ γ,λ,

where lim_*γ*→*∞*_⁡ Mode(*X*∣*γ*, *λ*)/*E*(*X*∣*γ*, *λ*) = 1.

Shannon's Entropy from EEG distribution [9], which played a central role as a measure of the uncertainty associated with a random variable, is given by

(10) HSϕ,λ,α=log⁡γλ+2log⁡γ−γ2log⁡γλ−1−2γ+2γ−1.

## 3. Methods of Estimation

In this section, we discuss ten different estimation methods to obtain the estimates of the parameters *γ* and *λ* of the EEG distribution.

### 3.1. Maximum Likelihood Estimation

Among the statistical inference methods, the maximum likelihood method is widely used due its desirable properties including consistency, asymptotic efficiency, and invariance. Under the maximum likelihood method, the estimators are obtained from maximizing the likelihood function (see e.g., [17]). Let *T*_1_,…, *T*_*n*_ be a random sample such that *T* ~ EEG(*γ*, *λ*); the likelihood function from (1) is given by

(11) Lγ,λ;x=∏i=1nfxi,γ,λ=λγnexp−λ∑i=1nxi∏i=1n1−1−γe−λxi−2.

The logarithm of the likelihood function (11) is given by

(12) lγ,λ ∣ x=nlog⁡λγ−λ∑i=1nxi−2∑i=1nlog⁡1−1−γe−λxi.

From ∂*l*(*γ*, *λ*∣**t**)/∂*γ* = 0 and ∂*l*(*γ*, *λ*∣**t**)/∂*λ* = 0, we get the likelihood equations

(13) nλ−∑i=1nxi−21−λ∑i=1nxie−λxi1−1−γe−λxi=0,

(14) nλ−2∑i=1ne−λxi1−1−γe−λxi=0,

whose solutions provide the maximum likelihood estimates, hereafter, *γ*_MLE_ and *λ*_MLE_. Numerical methods such as Newton-Raphson are required to find the solution of the nonlinear system.

For large sample sizes, the obtained estimators are not biased and asymptotically efficient. The MLE estimates are asymptotically normally distributed with joint bivariate normal distribution given by

(15) γ^MLE,λ^MLE~N2γ,λ,I−1γ,λfor  n⟶∞,

where *I*(*γ*, *λ*) is the Fisher information matrix given by (see [14])

(16) Iγ,λ=I11γ,λnγ−1−γ2 logγ3γλγ−12nγ−1−γ2log⁡γ3γλγ−12n3γ2,

(17) I11γ,λ=n31−γ−21−γ−γL21−γ3λ21−γif  0<γ<1n3λ21−γ1−γ1+π23+log2γ−2L21γ,if  γ>1.

### 3.2. Moments Estimators

The method of moments is one of the oldest procedures used for estimating parameters in statistical models. The moment estimators (ME) of the EEG distribution can be obtained by equating the first two theoretical moments,

(18) 1n∑i=1nxi=γlog⁡γλγ−1,1n∑i=1nxi2=2γL21−γλ21−γ,

with the sample moments $\bar{x}=\left(1/n\right)\sum_{i=1}^{n}x_{i}$ and (1/*n*)∑_*i*=1_^*n*^ *x*_*i*_^2^, respectively. After some algebraic manipulation, the estimate for ${\hat{\lambda }}_{ME}$ can be obtained by solving

(19) $$\begin{array}{c} {\hat{\lambda }}_{ME}=\frac{\gamma \log\left(\gamma \right)}{\bar{x}\left(\gamma -1\right)}. \end{array}$$

Note that, by substituting ${\hat{\lambda }}_{ME}$ in (18), the estimate for ${\hat{\gamma }}_{ME}$ can be obtained by solving

(20) $$\begin{array}{c} \frac{2\left(1-\gamma \right)L_{2}\left(1-\gamma \right){\bar{x}}^{2}}{\gamma \log{\left(\gamma \right)}^{2}}-\frac{1}{n}\overset{n}{\underset{i=1}{\sum }}x_{i}^{2}=0. \end{array}$$

Therefore, we firstly compute ${\hat{\gamma }}_{ME}$ and, by substituting such estimate in (19), the estimate ${\hat{\lambda }}_{ME}$ is obtained.

### 3.3. Method of Modified Moments

A simple modification can be made in the method of moments for estimating the parameters of the EEG distribution. To obtain the moment estimators (MME), consider that

(21) EX ∣ γ,λ=γlog⁡γλγ−1,VarX ∣ γ,λ=γλ22L21−γ1−γ−γ log2⁡γ1−γ2.

Note that the population coefficient of variation given by

(22) CVX ∣ γ,λVarX ∣ γ,λEX ∣ γ,λ=2γ−1−1L21−γlog2⁡γ−1

is independent of the scale parameter *λ*. So the estimate for ${\hat{\gamma }}_{ME}$ can be obtained by solving the nonlinear equation

(23) $$\begin{array}{c} \sqrt{\frac{2\left(\gamma^{-1}-1\right)L_{2}\left(1-\gamma \right)}{\log^{2}\left(\gamma \right)}-1}-\frac{s}{\bar{x}}=0 \end{array}$$

and, by substituting ${\hat{\gamma }}_{MME}$ in (23), the estimate ${\hat{\lambda }}_{MME}$ for *λ* can be obtained by solving

(24) $$\begin{array}{c} {\hat{\lambda }}_{MME}=\frac{\gamma \log\left(\gamma \right)}{\bar{x}\left(\gamma -1\right)}. \end{array}$$

### 3.4. Percentile Estimators

The percentile estimator, originally suggested by Kao [18, 19], is a statistical method used to estimate the unknown parameters by comparing the sample points with the theoretical ones. This method has been widely used in distributions that have the quantile function in a closed form, such as the Weibull distribution and the Generalized Exponential distribution. For the EEG distribution, the quantile function is given by

(25) $$\begin{array}{c} Q\left(\;p\;|\;\gamma ,\lambda \;\right)=\frac{1}{\lambda }log\left(\;\frac{1-\left(1-\gamma \right)p}{1-p}\;\right). \end{array}$$

The percentile estimates (PCE), ${\hat{\gamma }}_{PCE}$ and ${\hat{\lambda }}_{PCE}$, can be obtained by minimizing

(26) $$\begin{array}{c} \overset{n}{\underset{i=1}{\sum }}{\left(\;x_{\left(i\right)}-\frac{1}{\lambda }log\left(\;\frac{1-\left(1-\gamma \right)p}{1-p}\;\right)\;\right)}^{2}, \end{array}$$

with respect to *γ* and *λ*, where *p*_*i*_ denotes some estimate of *F*(*x*_(*i*)_; *γ*, *λ*). The estimates of *γ* and *λ* can also be obtained by solving the following nonlinear equations:

(27) ∑i=1nxi−1λlog1−1−γp1−pp1−1−γp=0,

(28) ∑i=1nxi−1λlog⁡1−1−γp1−p·1λ2log⁡1−1−γp1−p=0,

respectively. In this paper, we consider *p*_*i*_ = *i*/(*n* + 1). However, several estimators of *p*_*i*_ can be used instead (see [20]).

### 3.5. *L*-Moments Estimators

Hosking [21] proposed an alternative method of estimation analogous to conventional moments, namely,* L*-moments estimators. These estimators are obtained by equating the sample* L*-moments with the population* L*-moments. Hosking [21] states that the* L*-moment estimators are more robust than the usual moment estimators and are also relatively robust to the effects of outliers and reasonably efficient when compared to the MLE for some distributions.

For the EEG distribution, the* L*-moments estimators (LME) can be obtained by equating the first two sample* L*-moments with the corresponding population* L*-moments. The first two sample* L*-moments are

(29) l1=1n∑i=1nxi,l2=2nn−1∑i=1ni−1xi−l1,

and the first two population* L*-moments are

(30) μ1γ,λ∫01Qp ∣ γ,λdp=EX ∣ γ,λ=γlog⁡γλγ−1,μ2γ,λ∫01Qp ∣ γ,λ2p−1dp=1λγ2−2γlog⁡γ−12γ−12+12,

where *Q*(*p*∣*γ*, *λ*) is given in (25). After some algebraic manipulations, the estimate for ${\hat{\gamma }}_{LME}$ can be obtained by solving the nonlinear equation

(31) $$\begin{array}{c} \frac{\bar{x}}{1-\gamma }+\frac{\bar{x}}{\log\left(\gamma \right)}-l_{2}=0. \end{array}$$

Note that, by substituting ${\hat{\gamma }}_{LME}$ in (30), the estimate for ${\hat{\lambda }}_{LME}$ can be obtained by solving

(32) $$\begin{array}{c} {\hat{\lambda }}_{LME}=\frac{{\hat{\gamma }}_{LME}\log\left({\hat{\gamma }}_{LME}\right)}{\bar{x}\left({\hat{\gamma }}_{LME}-1\right)}. \end{array}$$

### 3.6. Ordinary and Weighted Least Squares Estimates

Let *t*_(1)_, *t*_(2)_,…, *t*_(*n*)_ denote the order statistics (we assume the same notation for the next subsections) of the random sample of size *n* from a distribution function *F*(**x**∣*γ*, *λ*). The least square estimators (LSE) ${\hat{\gamma }}_{LSE}$ and ${\hat{\lambda }}_{LSE}$ can be obtained by minimizing

(33) $$\begin{array}{c} S\left(\;\gamma ,\lambda \;\right)=\overset{n}{\underset{i=1}{\sum }}{\left[\;F\left(\;x_{\left(i\right)}\;|\;\gamma ,\lambda \;\right)-\frac{i}{n+1}\;\right]}^{2}, \end{array}$$

with respect to *γ* and *λ*, where *F*(**t** | *γ*, *λ*) is given by (2). Equivalently, they can be obtained by solving the following nonlinear equations:

(34) ∑i=1nFxi ∣ γ,λ−in+1Δ1xi ∣ γ,λ=0,∑i=1nFxi ∣ γ,λ−in+1Δ2xi ∣ γ,λ=0,

where

(35) Δ1xi ∣ γ,λ=eλxi−1eλxi−1+γ2,Δ2xi ∣ γ,λ=λxeλxieλxi−1+γ2.

The weighted least squares estimates (WLSE), ${\hat{\gamma }}_{WLSE}$ and ${\hat{\lambda }}_{WLSE}$, can be obtained by minimizing

(36) Wγ,λ=∑i=1nn+12n+2in−i+1Fti ∣ γ,λ−in+12.

These estimates can also be obtained by solving the following nonlinear equations:

(37) ∑i=1nn+12n+2in−i+1Fxi ∣ γ,λ−in+1·Δ1xi ∣ γ,λ=0,∑i=1nn+12n+2in−i+1Fxi ∣ γ,λ−in+1·Δ2xi ∣ γ,λ=0.

### 3.7. Method of Maximum Product of Spacings

The maximum product of spacings (MPS) method is a powerful alternative to MLE for the estimation of the unknown parameters of continuous univariate distributions. Proposed by Cheng and Amin [22, 23], this method was also independently developed by Ranneby [24] as approximation to the Kullback-Leibler measure of information.

Let *D*_*i*_(*γ*, *λ*) = *F*(*x*_(*i*)_∣*γ*, *λ*) − *F*(*x*_(*i*−1)_∣*γ*, *λ*), for *i* = 1,2,…, *n* + 1, be the uniform spacings of a random sample from the EEG distribution, where *F*(*x*_(0)_∣*γ*, *λ*) = 0 and *F*(*x*_(*n*+1)_∣*γ*, *λ*) = 1. Clearly ∑_*i*=1_^*n*+1^ *D*_*i*_(*γ*, *λ*) = 1. The maximum product of spacings estimates, ${\hat{\gamma }}_{MPS}$ and ${\hat{\lambda }}_{MPS}$, are obtained by maximizing the geometric mean of the spacings,

(38) $$\begin{array}{c} G\left(\;\gamma ,\lambda \;\right)={\left[\;\prod_{i=1}^{n+1}D_{i}\left(\;\gamma ,\lambda \;\right)\;\right]}^{1/\left(n+1\right)}, \end{array}$$

with respect to *γ* and *λ*, or, equivalently, by maximizing the logarithm of the geometric mean of sample spacings:

(39) $$\begin{array}{c} H\left(\;\gamma ,\lambda \;\right)=\frac{1}{n+1}\overset{n+1}{\underset{i=1}{\sum }}\log D_{i}\left(\;\gamma ,\lambda \;\right). \end{array}$$

The estimates ${\hat{\gamma }}_{MPS}$ and ${\hat{\lambda }}_{MPS}$ of the parameters *γ* and *λ* can be obtained by solving the following nonlinear equations

(40) ∂Hγ,λ∂γ=1n+1·∑i=1n+11Diγ,λΔ1xi ∣ γ,λ−Δ1xi−1 ∣ γ,λ=0,∂Hγ,λ∂λ=1n+1·∑i=1n+11Diγ,λΔ2xi ∣ γ,λ−Δ2xi−1 ∣ γ,λ=0,

where Δ_1_(·∣*γ*, *λ*) and Δ_2_(·∣*γ*, *λ*) are given in (35).

Note that if *x*_(*i* + *k*)_ = *x*_(*i* + *k* − 1)_ = ⋯ = *x*_(*i*)_ we get *D*_*i*+*k*_(*γ*, *λ*) = *D*_*i*+*k*−1_(*γ*, *λ*) = ⋯ = *D*_*i*_(*γ*, *λ*) = 0. Therefore, the MPS estimators are sensitive to closely spaced observations, especially ties. When the ties are due to multiple observations, *D*_*i*_(*γ*, *λ*) should be replaced by the corresponding likelihood *f*(*x*_(*i*)_, *γ*, *λ*), since *x*_(*i*)_ = *x*_(*i* − 1)_.

Cheng and Amin [23] proved desirable properties of the MPS such as asymptotic efficiency and invariance; they also proved that the consistency of maximum product of spacings estimators holds under much more general conditions than for maximum likelihood estimators. The authors also present an interesting proof that the MPS estimates converge asymptotically to the ML estimates. Therefore, for the EEG distribution, the MPS estimators are asymptotically normally distributed (see [25] for more details) with joint bivariate normal distribution given by

(41) γ^MPS,λ^MPS~N2γ,λ,I−1γ,λfor  n⟶∞,

where *I*(*γ*, *λ*) is the Fisher information matrix.

### 3.8. The Cramer-von Mises Minimum Distance Estimators

The Cramer-von Mises estimator (CME) is a type of minimum distance estimators (also called maximum goodness-of-fit estimators) which is based on the difference between the estimate of the cumulative distribution function and the empirical distribution function (see, [26, 27]).

MacDonald [28] motivates the choice of Cramer-von Mises type minimum distance estimators providing empirical evidence that the bias of the estimator is smaller than the other minimum distance estimators. The Cramer-von Mises estimates, ${\hat{\gamma }}_{CME}$ and ${\hat{\lambda }}_{CME}$, are obtained by minimizing

(42) $$\begin{array}{c} C\left(\;\gamma ,\lambda \;\right)=\frac{1}{12n}+\overset{n}{\underset{i=1}{\sum }}{\left(\;F\left(\;x_{\left(i\right)}\;|\;\gamma ,\lambda \;\right)-\frac{2i-1}{2n}\;\right)}^{2}, \end{array}$$

with respect to *γ* and *λ*. These estimates can also be obtained by solving the following nonlinear equations:

(43) ∑i=1nFxi ∣ γ,λ−2i−12nΔ1xi ∣ γ,λ=0,∑i=1nFxi ∣ γ,λ−2i−12nΔ2xi ∣ γ,λ=0,

where Δ_1_(·∣*γ*, *λ*) and Δ_2_(·∣*γ*, *λ*) are given in (35).

### 3.9. Methods of Anderson-Darling

Another type of minimum distance estimators is based on Anderson-Darling statistic (see [27]) and is known as the Anderson-Darling estimator (ADE). The Anderson-Darling estimates, ${\hat{\gamma }}_{ADE}$ and ${\hat{\lambda }}_{ADE}$, of the parameters *γ* and *λ* are obtained by minimizing, with respect to *γ* and *λ*, the function

(44) Aγ,λ=−n−1n∑i=1n2i−1·log⁡Fxi ∣ γ,λ+log⁡Sxn+1−i ∣ γ,λ.

These estimates can also be obtained by solving the following nonlinear equations:

(45) ∑i=1n2i−1Δ1xi ∣ γ,λFxi ∣ γ,λ−Δ1xn+1−i ∣ γ,λSxn+1−i ∣ γ,λ=0,∑i=1n2i−1Δ2xi ∣ γ,λFxi ∣ γ,λ−Δ2xn+1−i ∣ γ,λSxn+1−i ∣ γ,λ=0,

where Δ_1_(·∣*γ*, *λ*) and Δ_2_(·∣*γ*, *λ*) are in (35).

## 4. Simulation Study

In this section, we develop a simulation study via Monte Carlo methods. The main goal of these simulations is to compare the efficiency of the different estimation methods for the parameters of the EEG distribution. The following procedure was adopted:

(1) Set the sample size *n* and the vector of parameter values ***θ*** = (*λ*, *γ*).
(2) Generate values of EEG(*λ*, *γ*) with size *n*.
(3) Using the values obtained in step (2), compute $\hat{\lambda }$ and $\hat{\gamma }$ via MLE, ME, MME, LSE, WLSE, PCE, MPS, CME, and ADE.
(4) Repeat steps (2) and (3) *N* times.
(5) Using $\hat{\theta }$ and ***θ***, compute the mean relative estimates (MRE) $\sum_{j=1}^{N}\left(\left({\hat{\theta }}_{i,j}/\theta_{i}\right)/N\right)$ and the mean square errors (MSE) $\sum_{j=1}^{N}\left({\left({\hat{\theta }}_{i,j}-\theta_{i}\right)}^{2}/N\right)$, *i* = 1,2.

We expect that, considering this approach, the MREs are closer to one with smaller MSEs. The results were computed using the software R (R Core Development Team). The seed used to generate the random values was 2015. The chosen values to perform this procedure were ***θ*** = ((0.5,2), (2, 4)), *N* = 10,000, and *n* = (15, 20, 25,…, 130). The values of ***θ*** were selected to allow, respectively, the decreasing and increasing shape in the hazard function. Another motivation comes from the fact that, for ***θ*** = (0.5,2), we have analogous results for the exponential geometric distribution [2] and, for ***θ*** = (2,4), the results are analogous for the Complementary Exponential Geometric distribution [4].

**Figure 2:**
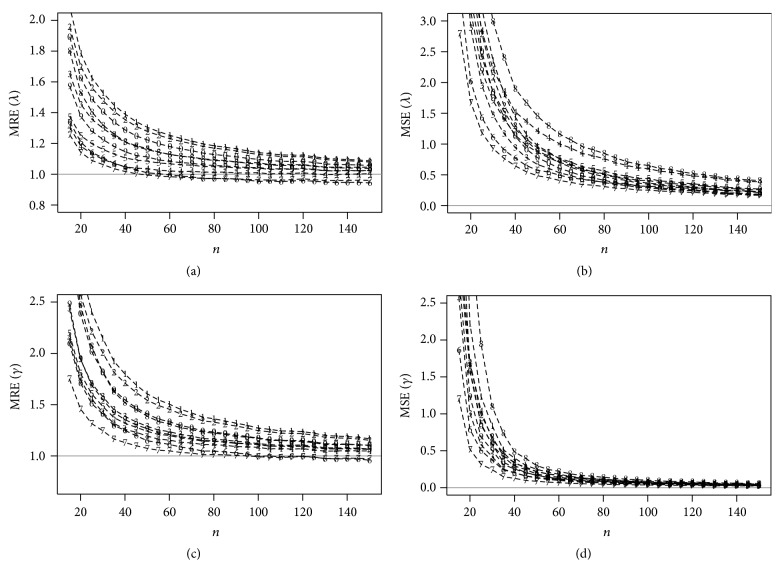
MREs and MSEs related from the estimates of *λ* = 2 and *γ* = 0.5 for *N* simulated samples, considering different values of *n* obtained using the following estimation methods: (1) MLE, (2) ME, (3) MME, (4) LME, (5) LSE, (6) WLSE, (7) PCE, (8) MPS, (9) CME, and (10) ADE.

**Figure 3:**
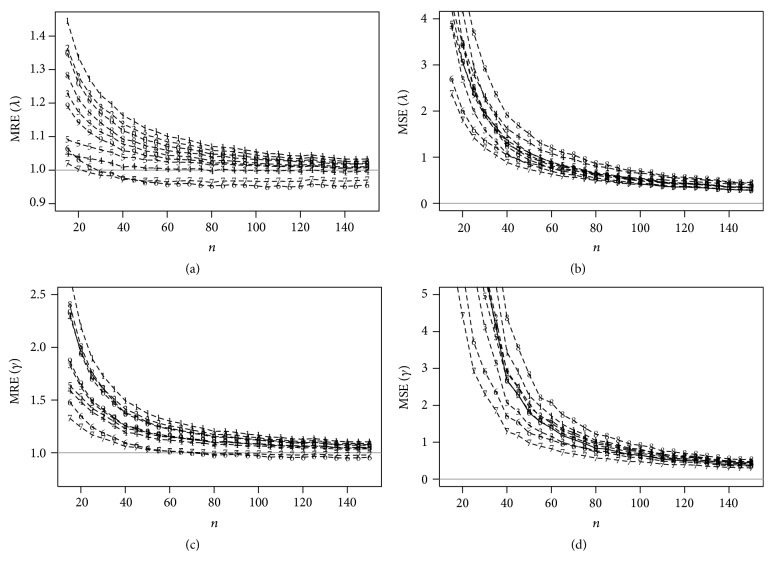
MREs and MSEs related from the estimates of *λ* = 4 and *γ* = 2 for *N* simulated samples, considering different values of *n* obtained using the following estimation methods: (1) MLE, (2) ME, (3) MME, (4) LME, (5) LSE, (6) WLSE, (7) PCE, (8) MPS, (9) CME, and (10) ADE.

Figures 2 and 3 present the MREs and MSEs for the estimates of ***θ*** for *N* simulated samples considering different values of *n*. The horizontal lines in Figures 2 and 3 correspond to MREs and MSEs being, respectively, one and zero.

**Figure 4:**
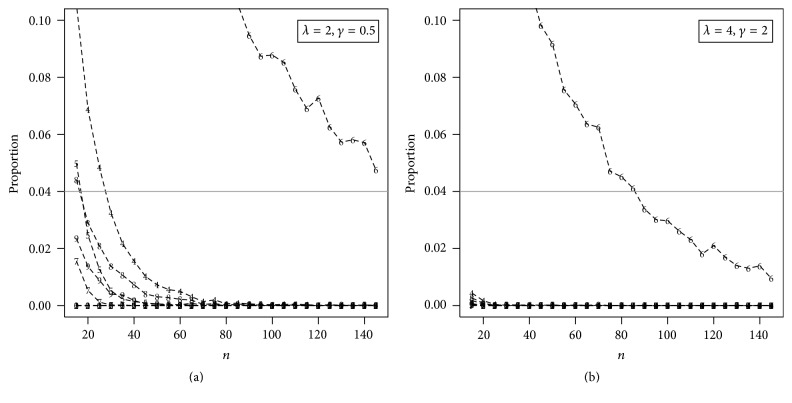
Rate of convergence considering different values of *n* obtained using the following estimation methods: (1) MLE, (2) ME, (3) MME, (4) LME, (5) LSE, (6) WLSE, (7) PCE, (8) MPS, (9) CME, and (10) ADE.

It is worth noting that we only considered the samples in which all estimation procedures had converged, getting at the end *N* simulated samples for different values of *n*.  Figure 4 presents the proportion of failure from each method.

Based on these figures, the MSEs of all estimates tend to zero for large *n* and also, as expected, the values of MREs tend to one; that is, the estimates are asymptotically unbiased for the parameters. The ME and the CME estimators have, respectively, the largest MREs and MSEs among all the considered estimators. The percentile and the LSE estimators have, respectively, the largest proportion of failure for estimating the parameters of the EEG distribution.

The MPS estimators have the smallest MSEs and the MREs nearest to one for both parameters proving to be the most efficient procedure for estimating the unknown parameters. Moreover, the MPS estimators have good theoretical properties [23] such as consistency, asymptotic efficiency, normality, and invariance. Therefore, we conclude that the MPS estimators should be used for estimating the parameters of the EEG distribution.

## 5. Applications

In this section, we considered two real data sets. The first one is presented by Boag [29] and is related to the ages (in months) of 18 patients who died from other causes than cancer. The second data set is presented by Silva [30] and refers to the serum-reversal time (in days) of 143 children born to HIV-infected mothers who did not receive anti-HIV treatment (Table 4).

In Section 4, our simulation study indicated that the MPS estimators should be used for estimating the parameters of the EEG distribution. Initially, we compared the estimates obtained from the different procedures with the MPS estimator in terms of MREs. Then, we compared the results obtained from the EEG distribution fitted by the MPS estimators with some common lifetime models, such as Weibull, Gamma, Lognormal, and Generalized Exponential distributions.

The Kolmogorov-Smirnov (KS) test is considered to check the goodness of fit. This procedure is based on the KS statistic *D*_*n*_ = sup_*x*_⁡|*F*_*n*_(*x*) − *F*(*x*; *θ*, *λ*)|, where sup⁡*x* is the supremum of the set of distances, *F*_*n*_(*x*) is the empirical distribution function, and *F*(*x*; *θ*, *λ*) is cumulative distribution function. In this case, we test the null hypothesis that the data comes from *F*(*x*; *θ*, *λ*), and, with significance level of 5%, we will reject the null hypothesis if *p* value is smaller than 0.05. As discrimination criterion method, we considered the AIC (Akaike Information Criteria), AICc (Corrected Akaike Information Criteria), HQIC (Hannan-Quinn Information Criteria), and the CAIC (Consistent Akaike Information Criteria) computed, respectively, by $AIC=-2l(\hat{\Theta },x)+2k$, AICc = AIC + 2*k*(*k* + 1)/(*n* − *k* − 1), $HQIC=-2l(\hat{\Theta },x)+2k\log\left(log(n)\right)$, and $CAIC=-2l(\hat{\Theta },x)+k\log\left(n\right)+1$, where* k* is the number of parameters to be fitted and $\hat{\Theta }$ is the estimate of Θ. Given a set of candidate models for* t*, the preferred model is the one which provides the minimum values.

### 5.1. Boag Data Set

Table 1 presents the data set related to the ages (in months) of 18 patients who died from other causes than cancer extracted from Boag [29], which considered the Lognormal distribution to describe such data.

Considering the MPS estimators, we obtain ${\hat{\lambda }}_{MPS}=0.02101$ and CI_95%_(*λ*) = (0.00618; 0.03583) and ${\hat{\gamma }}_{MPS}=2.46430$ and CI_95%_(*γ*) = (0.00000; 6.28060). In Table 2, we compared the estimates obtained from the different procedures with the MPS estimator in terms of MREs.

**Figure 5:**
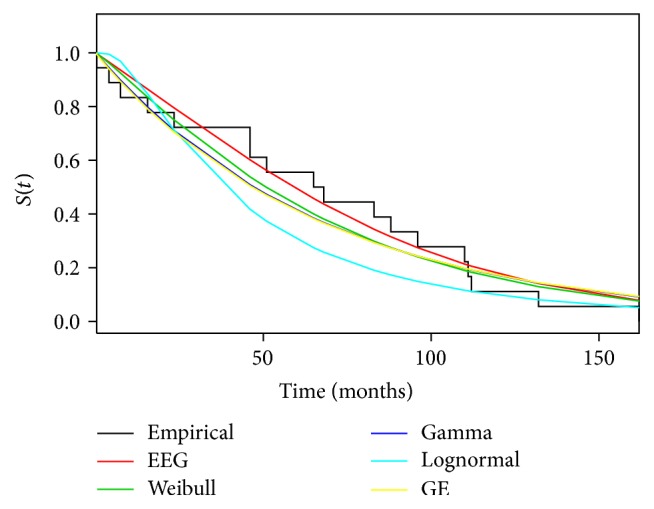
Survival function adjusted by different distributions and a nonparametric method considering the data sets related to the ages of 18 patients who died from other causes than cancer.

Table 2 confirmed the results obtained from our simulation study, in which for small sample sizes the obtained results may differ depending on the estimation procedure. For example, considering the method of moments, the estimate for *γ* is 52% smaller than ${\hat{\gamma }}_{MPS}$.  Table 3 presents the results from KS test (*p* value), AIC, AICc, HQIC, and CAIC, for the EEG distribution adjusted by the MPS procedure and for different probability distributions. In Figure 5, we have the survival function adjusted by different distributions and nonparametric survival estimator.

Comparing the empirical survival function with the adjusted distributions, a better fit for the EEG distribution among the chosen models can be observed. This result is confirmed from AIC, AICc, HQIC, and CAIC, since EEG distribution has the minimum values and *p* values returned from the KS test are greater than the chosen models. From our proposed methodology, we observe that the extended exponential geometric distribution has superior fit among the chosen models. In this case, each of the causes of the death can be described by exponential distribution; since the lifetime associated with a particular risk is not observable (latent variables), we observe only the maximum lifetime (*γ* > 1) value among all risks, where the number of causes follows geometric distribution.

### 5.2. Children Exposed to the Vertical Transmission of HIV

The data set related to the serum-reversal time (in days) of 143 children born to HIV-infected mothers is presented in Table 3.

Considering the MPS estimators, we obtain ${\hat{\lambda }}_{MPS}=0.0065$ and CI_95%_(*λ*) = (0.0054; 0.0077) and ${\hat{\gamma }}_{MPS}=14.2279$ and CI_95%_(*λ*) = (5.6714; 22.7843). In Table 5, we compared the estimates obtained from the different procedures with the MPS estimator in terms of MREs.

**Figure 6:**
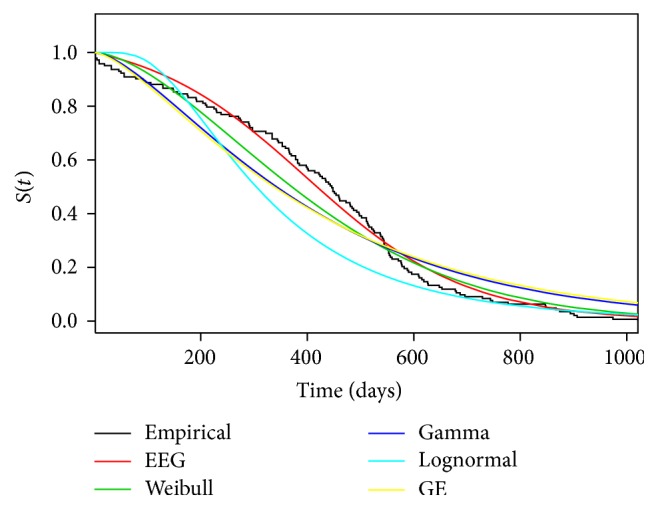
Survival function adjusted by different distributions and a nonparametric method considering the data sets related to the serum-reversal time (in days) of 143 children born to HIV-infected mothers.

From Table 5, we observed that for large samples sizes the estimates are very closer independently of the chosen method. Moreover, due to the large sample size, the MPS estimates and ML estimates are almost the same; such theoretical result is well supported by Cheng and Amin [23]. In Table 6, we have the results from KS test (*p* value), AIC, AICc, HQIC, and CAIC, for different probability distributions.  Figure 6 presents the survival function adjusted by different distributions and nonparametric survival estimator.

Comparing the empirical survival function with the adjusted distributions, a better fit for the extended exponential geometric distribution among the chosen models can be observed. This result is confirmed from AIC, AICc, HQIC, and CAIC, since EEG distribution has the minimum values among the chosen models. Moreover, considering a significance level of 5%, the EEG distribution was the only model in which *p* values returned from the KS test were greater than 0.05.

## 6. Conclusions

In this paper, we derived and compared, via intensive simulation study, the estimations of the parameters of the EEG distribution using ten estimation methods. Most importantly, from our simulations, we discovered that the estimates are asymptotically unbiased for the parameters regardless of the estimation method. However, while the ME and CME estimators have, respectively, the largest MREs and MSEs among all the considered estimators, the MPS estimator has the smallest MSEs and the MREs nearest to one, for both parameters, proving to be the most efficient method compared to others for estimating the unknown parameters. As a final advise, combining these results with the good properties of the method such as consistency, asymptotic efficiency, normality, and invariance, we conclude that the MPS estimator is the best one for estimating the parameters of the EEG distribution in comparison with its competitors. Finally, we apply our proposed methodology in two important data sets, demonstrating that the EEG distribution is a simple alternative to be used for lifetime applications.

## Figures and Tables

**Table 1:** Data set related to the ages of 18 patients who died from other causes than cancer.

0.3	4	7.4	15.5	23.4	46	46	51	65
68	83	88	96	110	111	112	132	162

**Table 2:** The MREs of the estimates obtained from the different procedures compared to the MPS considering the data set related to the ages of 18 patients who died from other causes than cancer.

${\hat{\theta }}_{MPS}/\hat{\theta }$	MLE	ME	MME	LME	LSE	WLSE	PCE	CME	ADE
*λ*	0.8033	0.7057	0.7397	0.8650	1.0365	0.9689	0.8192	0.8966	1.0112
*γ*	0.6281	0.4883	0.5537	0.8226	0.9630	0.8908	0.6186	0.7219	1.0222

**Table 3:** Results of the KS test (*p* value), AIC, AICc, HQIC, and CAIC for the different probability distributions considering the data set related to the ages of 18 patients who died from other causes than cancer.

Test	EEG	Weibull	Gamma	Lognormal	GE
KS	**0.9303**	0.5185	0.3361	0.0561	0.3156
AIC	**189.809**	191.447	191.801	201.695	191.798
AICc	**190.609**	192.247	192.601	202.495	192.598
HQIC	**190.054**	191.692	192.047	201.940	192.044
CAIC	**193.589**	195.227	195.582	205.475	195.579

**Table 4:** Data set related to the serum-reversal time (in days) of 143 children born to HIV-infected mothers.

2	2	2	5	9	9	19	32	32	46	50	56	56	78	91	95
106	129	129	148	149	156	175	176	191	192	204	209	211	225	229	230
238	254	271	274	276	290	291	292	297	297	322	334	334	334	344	346
353	353	359	365	366	367	370	378	378	382	382	385	398	400	402	414
422	424	428	434	435	440	443	446	448	448	451	454	459	460	461	473
480	481	484	487	493	497	498	502	511	511	513	514	516	521	524	526
537	538	541	543	544	544	545	549	551	553	553	554	556	559	571	576
577	578	582	588	590	596	609	610	615	619	626	627	648	653	678	680
687	696	729	744	748	777	847	848	867	874	894	901	907	974	1021	

**Table 5:** The MREs of the estimates obtained from the different procedures compared to the MPS considering the data set related to the serum-reversal time (in days) of 143 children born to HIV-infected mothers.

${\hat{\theta }}_{MPS}/\hat{\theta }$	MLE	ME	MME	LME	LSE	WLSE	PCE	CME	ADE
*λ*	0.9922	0.9726	0.9774	1.0007	0.9481	0.9587	0.9580	0.9352	1.0259
*γ*	0.9910	1.0169	1.0341	1.1184	0.8039	0.8684	0.9253	0.7682	1.1007

**Table 6:** Results of the KS test (*p* value), AIC, AICc, HQIC, and CAIC for the different probability distributions considering the data set related to the serum-reversal time (in days) of 143 children born to HIV-infected mothers.

Test	EEG	Weibull	Gamma	Lognormal	GE
KS	**0.6413**	0.0067	0.0000	0.0000	0.0000
AIC	**1950.82**	1981.31	2001.92	2088.90	2005.43
AICc	**1950.91**	1981.39	2002.01	2088.98	2005.52
HQIC	**1953.23**	1983.71	2004.33	2091.31	2007.84
CAIC	**1958.75**	1989.23	2009.85	2096.82	2013.36
